# Species‐ and site‐specific impacts of an invasive herbivore on tree survival in mixed forests

**DOI:** 10.1002/ece3.2002

**Published:** 2016-02-24

**Authors:** E. Penelope Holland, Andrew M. Gormley, Roger P. Pech

**Affiliations:** ^1^ Landcare ResearchPO Box 69040Lincoln7640New Zealand; ^2^ Department of BiologyUniversity of YorkHeslingtonYorkYO10 5DDUK

**Keywords:** Brushtail possum, herbivore management, herbivory models, plant–herbivore interactions, *Trichosurus vulpecula*

## Abstract

Invasive herbivores are often managed to limit their negative impact on plant populations, but herbivore density – plant damage relationships are notoriously spatially and temporally variable. Site and species characteristics (both plant and herbivore) must be considered when assessing the potential for herbivore damage, making it difficult to set thresholds for efficient management. Using the invasive brushtail possum *Trichosurus vulpecula* in New Zealand as a case study, we parameterized a generic model to predict annual probability of browse‐induced mortality of five tree species at 12 sites. We compared predicted and observed tree mortality for each species + site combination to establish herbivore abundance – tree mortality thresholds for each site on a single and combined tree species basis. Model results indicated it is likely that possum browse was the primary cause of all tree mortality at nine of the 12 species‐site combinations, allowing us to estimate site‐specific thresholds below which possum population numbers should be reduced and maintained to keep tree mortality under a predetermined level, for example 0.5% per year. The browse model can be used to set site‐ and species‐specific management action thresholds, and can be adapted easily for other plant or herbivore species. Results for multiple plant or herbivore species at a single site can be combined to create conservative, site‐wide management strategies, and used to: determine which sites will be affected most by changes in herbivore abundance; quantify thresholds for herbivore management; and justify expenditure on herbivore control.

## Introduction

Impacts of invasive herbivores on native biodiversity and agriculture are a significant component of ongoing global change (Mack et al. 2000). Herbivore management is often required to keep populations below thresholds where their damage is limited to acceptable levels. However, this can produce idiosyncratic outcomes: relationships between herbivore density and plant damage or mortality (see Hone 2007; Norbury et al. 2015) are notoriously variable, both spatially and temporally (Bee et al. 2009; Duncan et al. 2011; Asner and Levick 2012; Silva et al. 2013). The lack of a “one‐size‐fits‐all” relationship means that site and species differences must be considered when planning herbivore control (Gormley et al. 2012; Young et al. 2013).

The issue is compounded because herbivore control in mixed forests may not produce a proportionate reduction in browse impacts on a single species, or equivalent reduction among different species (Kamler et al. 2010). Nevertheless, management objectives are still expressed in terms of protecting vulnerable tree species or maintaining community‐scale forest composition (Norbury et al. 2015). Managers are therefore faced with the difficult question of how to achieve their objectives by distributing limited resources among multiple sites.

This problem is illustrated by the threat to native New Zealand forests posed by the brushtail possum *Trichosurus vulpecula* (Kerr), a nocturnal and omnivorous marsupial. Possums were introduced to New Zealand in the 19th century to establish a fur trade and spread rapidly, achieving high densities (c. 10/ha) in many podocarp/broadleaf forested areas in the absence of control (Clout and Ericksen 2000). Possums are primarily folivores, browsing on a wide range of tree species, with canopy species such as *Weinmannia racemosa, W. silvicola,* and *Beilschmiedia tawa* and some smaller tree species such as *Schefflera digitata* and *Olearia rani* forming a large part of the foliar diet (Nugent et al. 2000).

A key conclusion of several impact studies (Pekelharing et al. 1998; Todd et al. 2008; Kamler et al. 2010; Gormley et al. 2012) is that browse on individual trees and species depends on what other food is locally available (i.e., is context dependent; Bee et al. 2009). As a result, damage thresholds for different tree species and tolerable levels of herbivore abundance must be tailored to individual sites. How to define meaningful thresholds for a specific site remains equivocal, especially if a new site only has data collected from a single visit. This question was addressed by Holland et al. (2013), who created a model (hereafter “the browse model”) for predicting single‐species tree mortality resulting from herbivore browse damage that incorporates foraging behavior and tree growth rates. It can be parameterized using the type of data typically collected when monitoring herbivore browse damage, with initial model conditions set using site data recorded during a single visit.

This study parameterizes individual‐tree foliar dynamics for five possum‐preferred tree species, initializes the browse model for 12 species + site combinations, compares the predicted and observed tree mortality for each species + site combination to ascertain whether observed tree mortality could be attributed to possum browse rather than other mortality factors, and establishes herbivore abundance and tree mortality thresholds for each site to guide efficient management of browse impacts.

## Methods

### Overview

The model for browse‐induced tree mortality has three components. We briefly describe submodels (1) for the dynamics of foliar mass on individual trees; (2) for browse on individual trees within a species at a site; and (3) relating tree mortality to foliar mass. See Holland et al. (2013) for detailed model development. We explain how to (4) parameterize the individual‐tree component for each species in the browse model; (5) set initial site conditions for the model and ascertain species+site browse parameters; and (6) use model results to provide input for management decisions.

#### Tree foliage dynamics

The rate of change of a tree's foliar mass depends on foliage production rate, losses due to all processes other than herbivore browse (e.g., abscission), and losses due to browse:

(1) $$\frac{d\tau }{dt}=\frac{1}{a}\left(1-\text{exp}\left(-e\tau \right)\right)-\frac{\tau }{a}-\frac{I}{B}.$$

The maximum attainable foliar mass of a nonbrowsed tree is *B* kg. The first term in equation (1) describes the rate of foliage production as a function of the proportion of attainable foliar mass that remains in the canopy (*τ*). The rate increases as foliar mass increases, toward a maximum production rate of 1/*a*, where *a* is the average leaf life span. The rate at which foliage production increases with foliar mass is governed by the “growth efficiency”, *e*. The second term (*τ*/*a*) describes a constant, nonherbivore‐related foliage loss rate, which in the absence of browse, balances foliage production, leaving the tree with *B* kg of foliage (when *τ *= 1). The third term describes the herbivore intake rate (*I* kg per unit time) from that tree, as a proportion of attainable foliar mass. *I* represents the combined intake rate of all herbivores from that tree; it is a function of herbivore abundance and foraging behavior (i.e., how the herbivore population distributes its browse amongst trees at the site level; see section Site data for using the browse model below). The tree can maintain a constant (equilibrium) foliar mass for herbivore intake rates below a threshold *I′*, where

(2) $$I^{\prime }=\frac{B}{ae}\left(e+\text{log}\left(\frac{1}{e}\right)-1\right).$$

Foliage cannot be maintained if intake rates are >*I′*, in which case the tree will ultimately die. In its current form, the model assumes that complete defoliation results in tree death (see Discussion for situations where this is not applicable). The intake threshold as a proportion of attainable foliar mass (*I′*/*B*) specifies the maximum proportion of the tree that can be consumed without it succumbing to defoliation, and can be used to compare the resilience of different tree species to herbivory.

If the tree is to die within a specified period, the rate of change of *τ* (equation (1)) for that period must be negative. Here we assume all rates are measured on an annual basis. In this case, a tree will die within the next year if intake rates are greater than a mortality threshold ℑ(*τ*):

(3) $$I\geq B\left(\frac{1}{a}-\frac{\text{exp}(-e\tau )}{a}-\frac{\tau }{a}+\tau \right)=ℑ\left(\tau \right),$$

i.e., the annual probability of mortality of a tree is the probability that the intake rate from that tree is >ℑ(*τ*).

#### Browse on trees of single species at a site

Site‐wide foraging by herbivores on trees of the species of interest at site *s* is *I*_s_. This is not modeled explicitly as a function of herbivore abundance, because the proportion of diet coming from a tree species may vary between sites with different forest compositions. Instead, during initialization of the model, *I*_s_ is fitted using browse data and compared to measured herbivore abundance (if available) to check for consistency.

The mean foliage intake rate from the *j*th tree at the *s*th site is a weighted proportion of *I*_s_:

(4) $$I_{\mathrm{sj}}=\frac{{\left(B_{\mathrm{sj}}\right)}^{y_{s}}}{\sum_{k=1}^{n}{\left(B_{\mathrm{sk}}\right)}^{y_{s}}}I_{s},$$

where *B*_sj_ is the attainable foliar mass of the *j*th tree at site *s*, and *y*_s_ describes the degree of preferential browse on large or small canopies for the species of interest at site *s*. When *y*_s_ = 0, browse on each tree is independent of its foliar mass. When *y*_s_ = 1, browse is proportional to the fraction of that species' foliar mass that each tree provides at the site (i.e., preference is driven by foliage availability). If *y*_s_ > 1, herbivores prefer larger trees over and above the proportion of foliar mass that trees of that species provide at the site. If 0 < *y*_s_ < 1, herbivores do preferentially browse larger trees of that species (i.e., the choice is not random) but less so than if the preference is driven entirely by foliage availability.

Intake rates may also differ among equal‐sized trees within a species. This variability is modeled using the negative binomial distribution, with mean intake rate *μ*_sj_ (related to *I*_sj_ by herbivore bite size, see below) and dispersion parameter *h*_s_. Large values of *h*_s_ equate to more uniform browse among same‐species trees of similar size; when *h*_s_ < 1, some trees will have no browse and if *h*_s_ ≪ 1, most trees of that species will show no browse and a few individuals will suffer very high intake rates.

A feeding event is defined as the average mass of foliage removed from a single leaf by a herbivore in one visit (i.e., one or more bites, up to whole‐leaf size, after which the herbivore moves to another leaf). The mean intake rate from the *j*th tree at site *s*,* I*_sj_ (equation (4)), can be transformed into the number of “feeding events” per unit time, *μ*_sj_, by dividing by feeding event size (*f* kg). The size of a feeding event is assumed to be characteristic of a tree species, and constant between sites and over time. For a site with a known distribution of canopy sizes, *μ*_sj_ can be estimated as

(5) $$\mu_{\mathrm{sj}}=\frac{B_{\mathrm{sj}}^{y_{s}}}{nE\left({\left(B_{\mathrm{sk}}\right)}^{y_{s}}\right)}\frac{I_{s}}{f},$$

where *n* is the number of trees per hectare for a particular species and *E*((*B*_sk_)^ys^) denotes the expected value of attainable foliar mass per hectare at site *s* raised to the power *y*_s_.

#### Relationship between tree mortality and foliar mass

The probability of the *j*th tree at the *s*th site experiencing an intake rate higher than the mortality threshold ℑ(*τ*) (equation (3)) can be calculated using the cumulative negative binomial distribution as

(6) $$\begin{array}{cc} \text{Prob}(j\text{th}\; & \text{tree at site}\;s\;\text{dies within a year}|\tau_{\mathrm{sj}}=\tau \;\text{at year start})\\ & =1-\text{Prob}\left(\mu_{\mathrm{sj}}<\frac{J(\tau )}{f}\right),\\ & =I_{p}\left(\frac{J(\tau )}{f}+1,h_{s}\right), \end{array}$$

where I_*p*_ is the regularized incomplete beta function and *p *= *h*_s_/(*h*_s_ + *μ*_sj_).

#### Parameters for individual trees

Data from two studies (Parkes et al. 2006; Nugent et al. 2010) were used to estimate parameters for *W. racemosa, O. rani, S. digitata,* and *B. tawa*, using repeated observations of tagged trees at 51 sites across New Zealand (total number of trees per species shown in Table 1).

**Table 1:** Parameter estimates for within‐tree foliage dynamics derived from data in Parkes et al. (2006), Nugent et al. (2000, 2010)

Species	Code	No. tagged trees	No. observations, including repeats	Mean time between observations (years)	Average leaf lifespan (*a* years)	Growth efficiency (*e*)	Intake threshold (*I'/B*)	Feeding event size (*f* kg dry weight)	Estimated proportion of total diet
*Weinmannia racemosa*	WEIRAC	3525	8008	1.9	4.53	16.6	0.170	3.3 × 10^−5^	0.08
*W. silvicola*	WEISIL	–	–	–	4.53	16.6	0.170	3.3 × 10^−5^	0.33
*Olearia rani*	OLERAN	490	1359	2.1	3.46	14.54	0.216	3.83 × 10^−5^	0.1
*Schefflera digitata*	SCHDIG	282	606	3.8	3.92	60.79	0.234	1.6 × 10^−4^	0.4
*Beilschmiedia tawa*	BEITAW	959	3074	2.0	4.29	8.79	0.149	2.33 × 10^−5^	0.33

The Foliage Cover Index (FCI) and Foliar Browse Index (FBI) were measured at each observation (Payton et al. 1999). FCI is the proportion of light occluded by leaves when looking up through the tree crown, and is recorded in 10% categories. FBI is the proportion of the tree's leaves that show evidence of browse, and is recorded in categories: 0 (no browse), 1 (light: ≤25% leaves browsed), 2 (moderate: 26–50%), 3 (heavy: 51–75%), and 4 (severe: >75% leaves browsed). Tree diameter at breast height (DBH) was measured in centimeters.

These data were converted to quantities used in the browse model. From Holland (2013),

(7) FCI=1−exp(−τL),

where *L* is the leaf area index (m^2^ of foliage per m^2^ of crown) of an unbrowsed tree. This gives a saturating function for the proportion of light occluded by leaves. When *τ* or *L* are small, few leaves overlap and FCI is low. If *τ* and *L* are both high enough, FCI tends to 1 (i.e., complete overlap of leaves).

The amount of browse is given by

(8) FBI=1−ω,

where *ω* is the proportion of attainable foliar mass (*B*) that remains in intact leaves not browsed by the herbivore of interest (in this case, possums). This depends on the herbivore's foraging strategy (i.e., whether it always eats leaf tips, directs browse equally toward browsed and unbrowsed leaves, etc.; see Holland et al. 2013).

We used an allometric relationship between DBH and attainable foliar mass *B* calculated by Richardson et al. (2009) for native New Zealand tree species:

(9) $$B=0.0406{\text{DBH}}^{1.53}.$$

Browse model parameters describing foliage growth (*a* and *e*) were fitted to repeat measurements of FCI and FBI from Parkes et al. (2006) and Nugent et al. (2010). For each tree species, consecutive pairs of observations of FCI on individual trees were used to calculate the rate of change of leaf area as

(10) $$\frac{dL}{dt}=\frac{\ln\left(L_{t}\right)-\ln\left(L_{0}\right)}{t},$$

where *L*_t_ = −ln(1−FCI_t_) and ln is the natural log (Holland 2013). We then used linear regression (*lm* in R 2.13, R Development Core Team 2010) to estimate parameters for the model

(11) $$\frac{dL}{dt}=c_{0b}+c_{1b}\ln\left(L_{0}\right)=r_{b}\ln\left(\frac{\tilde{L_{b}}}{L_{0}}\right).$$

where *b *=* *0, 1, 2, 3, 4 denotes FBI category at time 0; that is, growth is assumed to depend on the initial leaf area index *L*_0_, mediated by current browse levels. Then *r*_b_ is the growth rate and $\tilde{L_{b}}$ is the asymptotic leaf area index of trees with browse *b*:* r*_b_ = −*c*_1*b*_ and

(12) $$\tilde{L_{b}}=\text{exp}\left(-\frac{c_{0b}}{c_{1b}}\right).$$

A random normal sample of 1000 simulated trees was generated for each browse category using the mean and standard deviation of model parameters *c*_0*b*_ and *c*_1*b*_. These were converted into growth rate and leaf area using equations (12) and (13). For each tree, the maximum value of $\tilde{L_{\mathrm{bj}}}$ (denoted $\tilde{L_{j}}$) across browse categories 0–2 was used to calculate $\tilde{\tau_{\mathrm{bj}}}=\tilde{L_{\mathrm{bj}}}/\tilde{L_{j}}$ (*j *=* *1…1000). Finally, we estimated growth parameters *a* and *e* using *nls* in R 2.13 to fit the model

(13) $$\tilde{r_{\mathrm{bj}}}=\frac{1}{a}\left(1-\text{exp}(-e\tilde{\tau_{\mathrm{bj}}})\right),$$

to the simulated data.

The final parameter required for each tree species is the size of a feeding event (*f*). Leaf masses (dry weight) were estimated from Richardson et al. (2004) and Salmon (1980). Based on photographs of browsed leaves of five tree species (including *W. racemosa* and *B. tawa*) in Payton et al. (1999), the size of a feeding event was assumed to be one‐quarter of the leaf for all species bar one (Table 1). The exception is *S. digitata*, for which a whole compound leaf is usually lost when possums browse on the petiole.

#### Site data for using the browse model

The browse model was applied to six sites in a management experiment by the New Zealand Department of Conservation to determine whether extensive control of possums could improve survival of possum‐preferred tree species (Gormley et al. 2012). Paired treatment (possum control) and nontreatment sites were used at three locations (Urewera, Haast and Coromandel). The treatment sites were chosen because they had all experienced regular possum control for at least a decade prior to this study, and the nontreatment sites selected to be similar in climate and environment to their paired treatment sites. At each site, >200 individual trees with DBH > 10 cm from two preferred tree species were tagged and observed for FCI, FBI, and DBH at Time 1 (either 2004 or 2006), and measured again at Time 2 (either 2009 or 2010) for survival, FCI and FBI. In total across all sites, five possum‐preferred tree species (*W. racemosa, W. silvicola, O. rani, S. digitata,* and *B. tawa*) were observed. Additional nonpreferred tree species were monitored to control for differing tree survival unrelated to browse effects (no differences found; see Gormley et al. 2012).

The number, DBH, FCI and species of all trees within 5‐m radius plots (98–137 plots per site) around a subset of tagged trees were measured at Time 2. Possum abundance was measured at each site at Times 1 and 2 using the trap‐catch index, with captures expressed as a percentage of trap‐nights (TCI; National Pest Control Agencies 2011). Bayesian hierarchical models were fitted to these data to determine a relationship between tree mortality and the parameters (FCI and DBH) that characterized tagged trees at the start of the 4‐ or 5‐year study period (Gormley et al. 2012).

*Weinmannia silvicola* was assumed to have the same tree‐specific parameters (*a, e*,* f*) as the closely related *W. racemosa*. Site‐level parameters describing tree species density, DBH distribution, possum intake rates and patterns of tree selection by possums within a 5‐m radius patch of forest were derived to initialize the browse model for a specific site, as follows. We used the mean number of trees recorded per species per 5‐m‐radius plot at Time 2 (from Gormley et al. 2012) to estimate the density (trees/ha) of each species at each site. A log‐normal distribution was fitted to DBH measurements of each species at each site at Time 1 (from Gormley et al. 2012) using *fitdistr* in R 2.13.

The site‐specific intake rate by possums could vary between sites but was assumed to be constant through time for each tree species. Therefore the model's predictions of browse impacts apply when continuous management holds the possum density at a fixed level or, if there is pulsed control, to intervals (e.g., 1 year) when the density is quasi‐stationary (see Discussion). We used FBI and DBH observations at Time 1 to fit species‐ and site‐specific values for total yearly intake rate (*I*_s_) and between‐tree variation parameters *y*_s_ and *h*_s_ as follows. For each tree of species *j* at site *s*, we estimated the attainable foliage (equation (9)), the total expected amount of foliage at the site (using the *y*_s_th moment of the tree size distribution) and hence the mean feeding rate (equation (5)) from each tree sampled at the site. We then calculated the probability of a zero (<1% browse damage recorded, allowing for observer error when actual browse is very low) or nonzero (>1% browse) FBI score using the negative binomial distribution with the estimated mean feeding rate for each tree, and *h*_s_. This was compared to the FBI data to find best‐fit values for *I*_s_, *y*_s_, and *h*_s_ using maximum‐likelihood (*optim* in R 2.13).

We transformed FCI observations for unbrowsed trees (Browse = 0) from Time 1 into leaf area indices using *L *= −log(1−FCI) (rearranging equation (7) with *τ *= 1). We calculated the mean leaf area index at each site, denoted *L*_s_, by fitting a normal distribution to the resulting set of *L* values, using *fitdistr* in R 2.13. These were used to translate model input and output from *τ* to FCI and vice versa, using equation (7).

#### Observed versus predicted tree mortality

Using equations (1), (2), (3), (4), (5), (6) and the mean and 95% confidence interval (95% CI) parameter values for *I*_s_, *y*_s_, and *h*_s_, we calculated the mean and 95% CI for the annual probability of tree mortality at each site by FCI category, and compared these to Gormley et al.'s (2012) empirical model for observed tree mortality. For each tree species, we also calculated the “site‐wide” annual probability of mortality, by weighting the browse model results in each FCI category by the proportion of trees in that category at Time 1. We converted these mortality rates into the equivalent number of trees in the samples at each site expected to die over the 4‐ or 5‐year period of the study, and compared these to the observed number of trees dying.

#### Managing browse damage for multiple tree species

Finally, the browse model was used to estimate the site‐wide annual probability of mortality for all species+site combinations for possum densities ranging from TCI = 0–50%. Although the trapping index will eventually saturate, the linear approximation between possum density and TCI is valid up to TCIs of ~50% (Ramsey et al. 2005), which is higher than any observed in this study. Site‐wide intake *I*_s_ was estimated per species + site as a function of TCI using the relationships

(14) TCI=3.88+3.96N,

(15) $$I_{s}=58.4Np_{\mathrm{diet}},$$

where *N* is possum density (Ramsey et al. 2005), annual intake per possum is equal to 58.4 kg/year (Nugent et al. 2000), and a conservative estimate of the proportion of annual intake by the whole possum population directed at each tree species (*p*_diet_) was taken from the largest percentage noted by Nugent et al. (2000). An arbitrary threshold of annual mortality = 0.5% (across all trees with DBH > 10 cm) was set to illustrate how the browse model can be used with a predetermined level acceptable to conservation managers. The TCI required to reduce mortality below that threshold was calculated for each species + site combination.

## Results

### Tree species parameters

Fitted values for average leaf lifespan (*a*) of the five tree species were 3.46–4.53 years and growth efficiency *e* ranged from 8.79 (*B. tawa*) to 60.79 (*S. digitata*), indicating different resilience of foliage production to browse or lowered foliar mass (Table 1). Higher values of *e* mean that foliage production is maintained close to the maximum growth rate (1/*a*) for longer as the proportion of attainable foliar mass (*τ*) is reduced.

The modeled annual intake thresholds for tree species' survival ranged from 15% (*B. tawa*) to 23% (*S. digitata*) of attainable foliar biomass (Table 1). It therefore appears that, of the tree species tested, *B. tawa* is the most susceptible to possum browse, while *S. digitata* is the most resilient. Uncertainty in tree species parameters is likely to mean that the results presented here are a conservative estimate (see Table S1).

### Site‐specific parameters

Densities of tree species ranged from 159 to 409 trees/ha at sites where the browse model was applied (Table 2). A preference for browse on larger trees was evident for 10 of the 12 species + sites (*y*_s_ > 0) (Table 2), with three of these displaying a preference in proportion to foliar mass (95% CI for *y*_s_ includes 1) and seven displaying a supra‐proportional preference in relation to foliar mass (95% CI for *y*_s_ > 1) (Table S1), consistent with the patterns observed in Gormley et al. (2012). The exceptions were *W. silvicola* at Coromandel (treatment and nontreatment sites), with *y*_s_ = 0.01. Browse among trees of similar size was extremely variable (*h*_s_ < 1) for 10 of the 12 species + sites. The exceptions were browse on *O. rani* at the Coromandel treatment site (*h*_s_ = 1.11), which resulted from very low browse on any trees of that species at that site, and *S. digitata* at the Haast treatment site (*h*_s_ = 3.54) (Table 2).

**Table 2:** Site‐specific parameters for the browse model (Holland et al. 2013)

	Site	TCI	Possums/ha	Plots per site	Species code	Tree density	*I*_s_	*y*_s_	*h*_s_	Mean DBH	*L*_s_
Coromandel	Treatment	2.0	<1	98	WEISIL	409	3.87	0.01	0.44	20.3	0.958
					OLERAN	231	0.01	3.61	1.11	17.1	0.988
	Nontreatment	47.4	11.0	125	WEISIL	318	672	0.01	0.13	18.4	0.986
					OLERAN	159	625	3.80	0.45	16.4	0.882
Haast	Treatment	3.4	<1	137	WEIRAC	320	11.7	1.76	0.02	35.9	1.113
					SCHDIG	159	4.32	1.00	3.54	12.7	0.842
	Nontreatment	21.4	4.4	136	WEIRAC	308	293	2.62	0.03	30.0	1.243
					SCHDIG	175	229	2.56	0.21	11.8	0.505
Urewera	Treatment	4.9	<1	106	WEIRAC	343	14.4	0.95	0.06	27.4	0.869
					BEITAW	333	30.1	1.75	0.11	28.8	1.094
	Nontreatment	35.4	8.0	100	WEIRAC	244	563	2.12	0.40	28.2	0.771
					BEITAW	293	451	1.79	0.31	27.4	1.306

Trap‐catch index (TCI) is a measure of possum population density. Tree density (trees/ha) includes only individuals with diameter at breast height (DBH, in cm) >10 cm. *I*_s_ is the total foliage intake rate (kg dry weight/ha/year) by possums at the *s*th site; *y*_s_ indicates the effect of tree size on possum foraging behavior at the *s*th site (negative values indicate a preference for smaller trees; positive, for larger trees); *h*_s_ indicates how selective (smaller *h*_s_) browse is among equally sized trees at the *s*th site; *L*_s_ is mean leaf area index (m^2^/m^2^ of foliage) of unbrowsed trees at the *s*th site.

The fitted values for intake rate of foliage by possums (*I*_s_) varied between tree species and sites (Tables 2 and S2). Total values of *I*_s_ per site were low (<45 kg/ha/year) for sites with low TCI (<5), and high (>522 kg/ha/year) for sites with high TCI (>20). Based on an average of 58.4 kg (digestible dry matter) per possum/year (Nugent et al. 2000), and assuming a diet composed entirely of foliage from the two tree species observed at each site, the low values of the total site intake correspond to possum densities <0.8/ha and the high total intake values correspond to >8.9 possums/ha. These estimates of possum densities are broadly consistent with trapping estimates: TCI = 4.9 (the highest of the “low” TCI sites) and TCI = 20.1–47.4 (the range of TCIs at the “high” sites, Table 2) equate to 0.25 and 4.4–11.0 possum/ha respectively (equation (14), Ramsey et al. 2005). However, the 95% CIs suggested for *I*_s_ were generally large (often one, and up to two orders of magnitude; Table S2), indicating high uncertainty over the amount of foliage being consumed, and possum diet is likely to include foliage from other tree species as well as the two observed in this study.

The DBH distribution of species at sites and average leaf area index (*L*_s_) of unbrowsed trees were variable among locations, but mostly similar between treatment and nontreatment sites at the same location (Table 2). Mean DBH ranged from 11.8 cm for the typically small species (*S. digitata* at Haast nontreatment) to 35.9 cm for large canopy species (*W. racemosa* at Haast treatment), although data were only measured for trees with DBH > 10 cm, which may bias the estimate upwards.

### Observed versus predicted tree mortality

**Figure 1:**
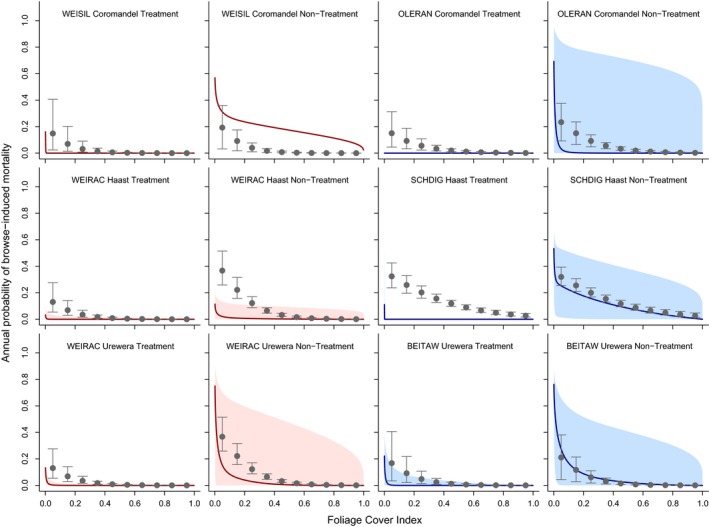
Annual probability of tree mortality attributable to possum browse predicted by the browse model (mean and 95% confidence intervals; solid line and shaded area respectively) as a function of Foliage Cover Index and compared with a hierarchical model of total mortality fitted to field data (mean and 95% confidence interval shown by gray circles and arrows; Gormley et al. 2012). *Weinmannia* in red and a second possum‐preferred species in blue. See Table 1 for full species names.

The browse model predicted a nonlinear relationship between FCI and tree mortality (Fig. 1). Predicted browse‐induced mortality was consistently lower at treatment than nontreatment sites and on treatment sites it was predicted to be close to zero except for trees in the lowest FCI category. The confidence intervals for browse‐induced mortality on nontreatment sites were much larger, due to wide confidence intervals for model parameters which are a result of uncertainty in data collection (see Table S2). The total mortality observed was within the 95% CI for predicted, browse‐induced mortality, which suggests that possum browse could have been responsible either directly or as an exacerbating factor alongside other causes of tree mortality for most mortality for all tree species observed at nontreatment sites (Fig. 1). Uncertainty in browse parameters did not qualitatively change the relationship produced (Fig. S1).

Taking into account the proportion of trees in each FCI category at Time 1, six of the 12 species + site combinations had observed mortality within the 95% CI for browse attributable to possums (Table 3) and three more had observed mortality close to that predicted, with values that would have required different survival of <5 trees (of samples of 213, 247, and 249 trees) to also fall within the 95% CI. The three remaining species + site combinations had considerably higher observed mortality than expected from possum browse alone.

**Table 3:** Predicted and observed annual probability of mortality of tree species at each site. “Difference” is the number of trees between the observed number that died in each sample (No. trees) and the 95% confidence interval for predicted deaths (if observed deaths do not fall within the interval). “TCI goal” is the trap‐catch index (TCI) of possum density that must be achieved to push site‐wide mortality confidence intervals below 0.005 (0.5% per year). TCI goals in bold are sites for which the acceptable limit for tree mortality was not met at the end of this study

Site	Code	No. trees	No. died	Annual mortality (%)	Predicted number of trees dying			Difference	TCI goal
					Lower	Mean	Upper		
Coromandel									
Treatment	WEISIL	213	3	0.283	0	0	0	3	49
	OLERAN	238	11	0.942	0	0	0	11	40
Nontreatment	WEISIL	225	2	0.178	1	4	41	–	**21**
	OLERAN	221	23	2.174	0	1	44	–	**22**
Haast									
Treatment	WEIRAC	247	5	0.408	0	0	1	4	19
	SCHDIG	197	94	12.16	0	0	0	94	18
Nontreatment	WEIRAC	229	0	0.000	0	1	14	–	**14**
	SCHDIG	200	114	15.53	3	45	135	–	**6**
Urewera									
Treatment	WEIRAC	237	1	0.106	1	1	1	–	22
	BEITAW	249	4	0.404	0	0	1	3	24
Nontreatment	WEIRAC	218	54	6.868	1	1	26	28	**17**
	BEITAW	229	3	0.329	1	1	7	–	**32**

### Managing browse damage for multiple tree species

**Figure 2:**
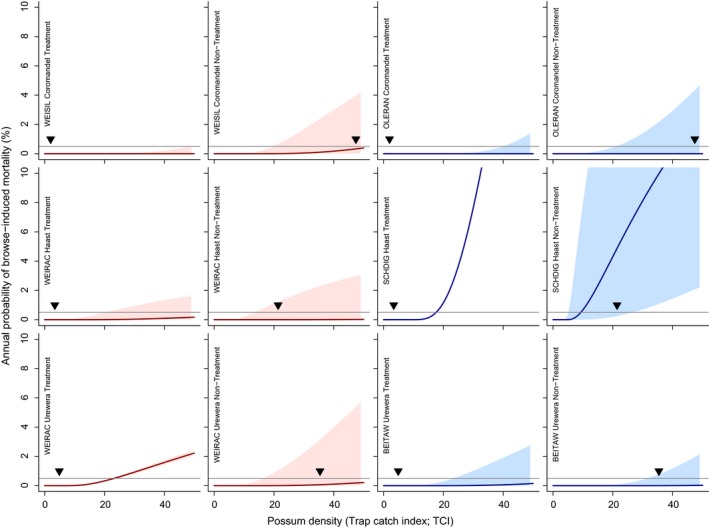
Mean and 95% confidence intervals for predicted site‐wide annual probability of mortality of species + site combinations as a function of possum density; solid line = mean, *Weinmannia* in red and a second possum‐preferred species in blue. The dotted line indicates site‐wide mortality equal to 0.5% per species per year, and the black triangle indicates the possum density of the site at Time 1.

Reducing possum density (TCI) at a site reduces the annual probability of tree mortality, but the relationship is nonlinear, and differences in foraging behavior mean that the benefits of herbivore control to a particular tree species varies between sites (Fig. 2). Tree mortality for all or a subset of preferred tree species at one site can be managed by identifying the lowest TCI required to maintain those species under the chosen mortality threshold (Fig. 2, Table 3). For example, protecting 95% of *S. digitata* trees, which is the second possum‐preferred species at Haast (Fig. 2), would require a TCI goal of 18 at the treatment site, but a TCI goal of six at the nontreatment site (the highest TCI for which the 95% confidence interval lies entirely below the threshold, Table 3). At both these sites, the TCI goal would also reduce possum density and hence browse to a level such that 95% of *W. racemosa* will survive.

## Discussion

We parameterized a browse model (Holland et al. 2013) for five possum‐preferred tree species, using data collected from two previous studies. Fitted values for average leaf lifespan of the five tree species were consistent with published values for evergreen, native New Zealand tree species including *W. racemosa* (Richardson et al. 2010), and fitted growth efficiencies make biological sense as *S. digitata* is fast‐growing relative to the other species in Table 1 (Forsyth et al. 2015). Independent data were used to compare predicted against observed mortality for five tree species in three new areas with paired possum treatment/nontreatment sites. The results indicate that it is likely possum browse caused mortality of trees with DBH > 10 cm at nine of the 12 species + site combinations. We estimated site‐specific thresholds below which possum numbers should be reduced and maintained to keep large‐tree mortality of multiple species under a predetermined level.

### Nonlinear negative relationship between FCI and mortality

All five tree species had a threshold foliar intake rate below which survival would be much reduced (Table 1). *Schefflera digitata* was the most resilient to possum browse, consistent with Gormley et al.'s (2012) hierarchical model showing that FCI (which is related to foliar mass) had the smallest negative effect on *S. digitata* tree mortality compared with the other species. The relationship between FCI and tree mortality seen in this study (Fig. 1) is consistent with those observed in Gormley et al. (2012).

### Herbivore preference for larger trees

Site parameters for the browse model indicated a tendency for possums to browse larger trees of all species observed (*y*_s_ **> 0; Table 2). This is consistent with the positive relationship between browse and tree size found by Gormley et al. (2012; the same data) and Duncan et al. (2011; independent data on possum browse of *W. racemosa*), indicating that large trees may provide an important food source for possums. Seven of the 12 species + site combinations showed evidence of strong selection for larger trees over and above the proportion of food that they provide at a site scale (95% CI of *y*_s_ entirely >1; see Supporting Information), suggesting other drivers for foraging preference may also be in play. The larger size of unbrowsed *W. racemosa* trees (i.e., higher leaf area and larger DBH) at Urewera compared to Haast could reflect different local environmental factors such as climate, topography, or soil conditions. The higher leaf area on *S. digitata* trees at Haast treatment (0.842) compared to Haast nontreatment (0.505) may be due to historical browse pressure being lower at the treatment site, allowing this fast growing species to recover quickly.

Herbivore preference for large trees is not limited to possums, having been recorded for koalas (Moore et al. 2010) and insects (Barrios 2003) among others. Given the global decline of large trees in forests at all latitudes (Lindenmayer et al. 2012), developing and using methods such as our browse model to identify sites and species at high risk from additional browse‐induced mortality of large trees should therefore be a management priority.

### Effect of herbivore control on tree mortality

Lower possum densities on treatment sites were consistent with predictions of reduced browse‐induced tree mortality on those sites, particularly for trees in higher FCI categories. As has been observed for other plant–herbivore interactions (e.g., Tremblay et al. 2007), the relationship between tree mortality and herbivore density (Fig. 2) is nonlinear and demonstrates the combined effect of differing palatability and resilience of tree species to herbivore browse. Overlaying these curves for multiple species at each site gives a simple way to assess site benefits of herbivore control. This approach allows managers to choose between setting an arbitrary target for herbivore control (which may not have the effect of maintaining forest composition) or explicitly managing for all or a subset of tree species present (by identifying the lowest TCI required to maintain those species under the chosen mortality threshold). For simplicity we assumed herbivore population density was quasi‐stationary, for example due to continuous management. Extension of the model to include herbivore population dynamics and a range of control scenarios is straightforward.

The mean predicted relationship between tree mortality and possum density was very similar for all tree species, when comparing between treatment and nontreatment sites in each area (Fig. 2). This should be expected, since the paired sites were chosen for their similar compositions and environmental attributes, and is an indicator that the model produces realistic output, especially given that Fig. 2 represents an extrapolation of results outside the single possum density (TCI) value recorded for each site. However, the confidence intervals around predicted tree mortality are much larger for nontreatment sites, most likely because the higher browse at those sites increases the potential range into which reasonable (and variable) herbivore intake rate, and foraging preference parameter values, may fall. A qualitatively similar pattern is seen when the confounding uncertainty of converting intake rates into possum densities is removed (Fig. S2).

### Applicability of the browse model to multiple tree species

For five of the six combinations of species and nontreatment sites, observed and predicted mortality were sufficiently similar to conclude that possum browse was the main factor reducing tree survival (Table 3). However, other factors can contribute to tree mortality (Bellingham et al. 1999), including at treatment sites where there were fewer possums and low or zero predicted deaths as a result of possum browse. Possum abundance is known to be patchy, and in some cases coincidence of localized high possum abundance within transects for tree monitoring could contribute to observed tree mortality being higher than predicted by the browse model for the wider area. This could explain the small discrepancies at the three sites that had fewer than five additional trees dying than was predicted, but is unlikely to be solely responsible for the 11 and 94 additional trees dying at Coromandel (*O. rani*) and Haast (*S. digitata*) treatment sites respectively. Additional factors are also likely to be causing mortality on *W. racemosa* at the Urewera nontreatment site, which had 28 more deaths than predicted. Clearly, browse‐induced mortality is less likely to mask mortality due to other factors on treatment sites with low possum density than on nontreatment sites.

### Applicability to additional tree species

The browse model can be adapted to any plant species where foliar mass is expected to affect growth rates and mortality (Holland et al. 2013). Parameterizing the model for additional tree species requires linking browse model components (e.g., *τ*,* ω*,* B*) to independent field data (e.g., FCI, FBI, DBH). Where specific data are not attainable, equivalent information may be used; Holland (2013) suggests FCI equivalents for assessing the impact of herbivory, disease, and pollution around the world, and Holland et al. (2013) cover a range of browse damage indices such as frequency/severity of damaged foliage, percentage of leaf area/stems missing, and height/stem‐based indices for browse on saplings by nonarboreal herbivores. Here, we assumed that tree death follows total defoliation, but this is clearly not the case for deciduous trees or those that can recover from complete defoliation. However, as long as the (net positive) foliage growth rate is lower than the (net negative) herbivore intake rate and leaf abscission rates combined during some range of foliar mass, then the qualitative behaviors of the browse model will be preserved (Holland et al. 2013).

### Applicability to other (and multiple) herbivores

The browse model can be adapted for other herbivores by changing the way browse is distributed within and among plants. Different options are explored in Holland et al. (2013), such as leaf tip browse, whole leaf removal, etc. Because browse is modeled as a series of feeding events by a population of herbivores, rather than specific browse patterns from individual herbivores, it is relevant for insect browse (smaller feeding events, with greater coverage of the canopy, e.g., Parsons et al. 2005) as well as browse by other marsupials (e.g., koalas; Todd et al. 2008) and larger mammals likely to remove entire shoots or branches (e.g., elephants; Makhabu et al. 2006), including those with targeted grazing tactics (e.g., deer; Kamler et al. 2009). The effect of concurrent browse by different herbivore species could be included via multiple intake rate terms in equation (1).

### Variable intake

We used a variable but size‐related approach to model the distribution of site‐wide foraging among trees, due to evidence that tree size is important in possum browsing preferences (Duncan et al. 2011; Gormley et al. 2012). However, the distribution of site‐wide intake among trees could be altered to depend on the behavioral ecology of different herbivores (e.g., ungulates preferring to browse in forest gaps; Kuijper et al. 2009). Once a distribution has been established to replace equations (4) and (5), the same approach for calculating plant mortality should apply (equation (6)). Modeling browse on multiple tree species, rather than each species in turn, would require a more complex model of the distribution of site‐wide intake but could provide insights into how herbivore browse affects trees in mixed forests.

### Management applications

Impacts of browse vary due to herbivore and plant population dynamics, differing community composition and subsequent foraging behavior. Site and species characteristics (both plant and herbivore) must therefore be considered when assessing potential for herbivore damage, and setting thresholds for browse impacts or herbivore density. Our browse model can be used to set site‐ and species‐specific management action thresholds in a relatively quick and inexpensive way because it can be parameterized using the type of data typically collected in herbivore monitoring studies (e.g., at minimum, indices of browse damage, canopy health and tree size, and some index of herbivore density), and calibrated for specific sites using single‐visit data. Moreover, results for multiple species at a single site can be combined for a conservative, site‐wide management strategy for pest control to reduce plant mortality. Because it can quantify the nonlinear relationships between herbivore density and browse damage on multiple species at different sites, the model facilitates a less conservative approach to herbivore management compared to a risk‐averse approach aimed at reducing herbivore density as much as possible at all sites: for example, some sites may not need any control despite having higher herbivore densities than others, due to the forest composition resulting in a more uniform browse impacts. Model outputs could be used to decide whether to pursue intervention based on a threshold *per se* or whether to operate routine control, and where control will generate the most benefit. In New Zealand forests, tree mortality is a result of multiple interacting processes, not just browse by invasive herbivores. Our results also indicate how much observed mortality is directly attributable to herbivore browse. All these factors are important for deciding whether or not management intervention is warranted at a particular site and for justifying expenditure on pest control.

## Data Accessibility

Data and R scripts are available at the Landcare Research repository at datastore.landcareresearch.co.nz and via http://dx.doi.org/10.7931/J29884X3.

## Conflict of Interest

None declared.

## Supporting information


**Figure S1.** Annual probability of tree mortality attributable to possum browse predicted by the browse model (mean and 95% confidence intervals indicating mortality of small, average and large trees; solid line and shaded area respectively) as a function of Foliage Cover Index, compared with an hierarchical model of total observed mortality fitted to field data (circles; Gormley et al. 2012), as per Fig. 1 in the main text.

**Figure S2.** Mean and 95% confidence intervals for predicted site‐wide annual probability of mortality of species + site combinations as a function of possum intake rate.

**Data S1.** Additional investigation of how uncertainty in tree species and site level parameters in the browse model will affect results. Includes Tables S1 and S2.
