# Supplementary figures and images for: Species‐ and site‐specific impacts of an invasive herbivore on tree survival in mixed forests
Source: Ecol Evol. 2016 Feb 24;6(7):1954–66. doi: 10.1002/ece3.2002 (PMC4767877; doi:10.1002/ece3.2002)

Annual probability of browse-induced mortality

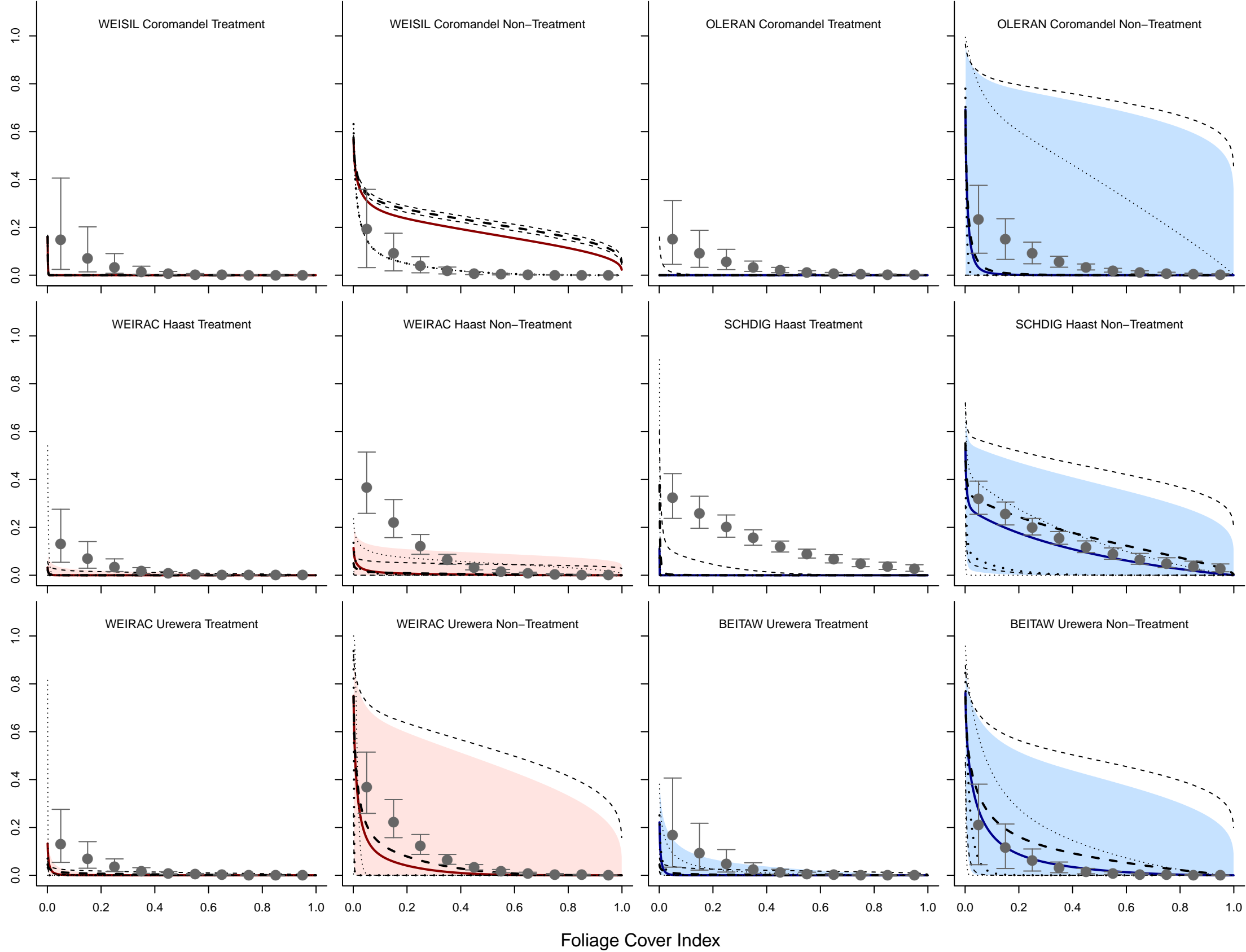

Supplement: Supplementary file 1 — Figure S1. Annual probability of tree mortality attributable to possum browse predicted by the browse model (mean and 95% confidence intervals indicating mortality of small, average and large trees; solid line and shaded area respectively) as a function of Foliage Cover Index, compared with an hierarchical model of total observed mortality fitted to field data (circles; Gormley et al. 2012), as per Fig. 1 in the main text. [file ECE3-6-1954-s001.pdf]

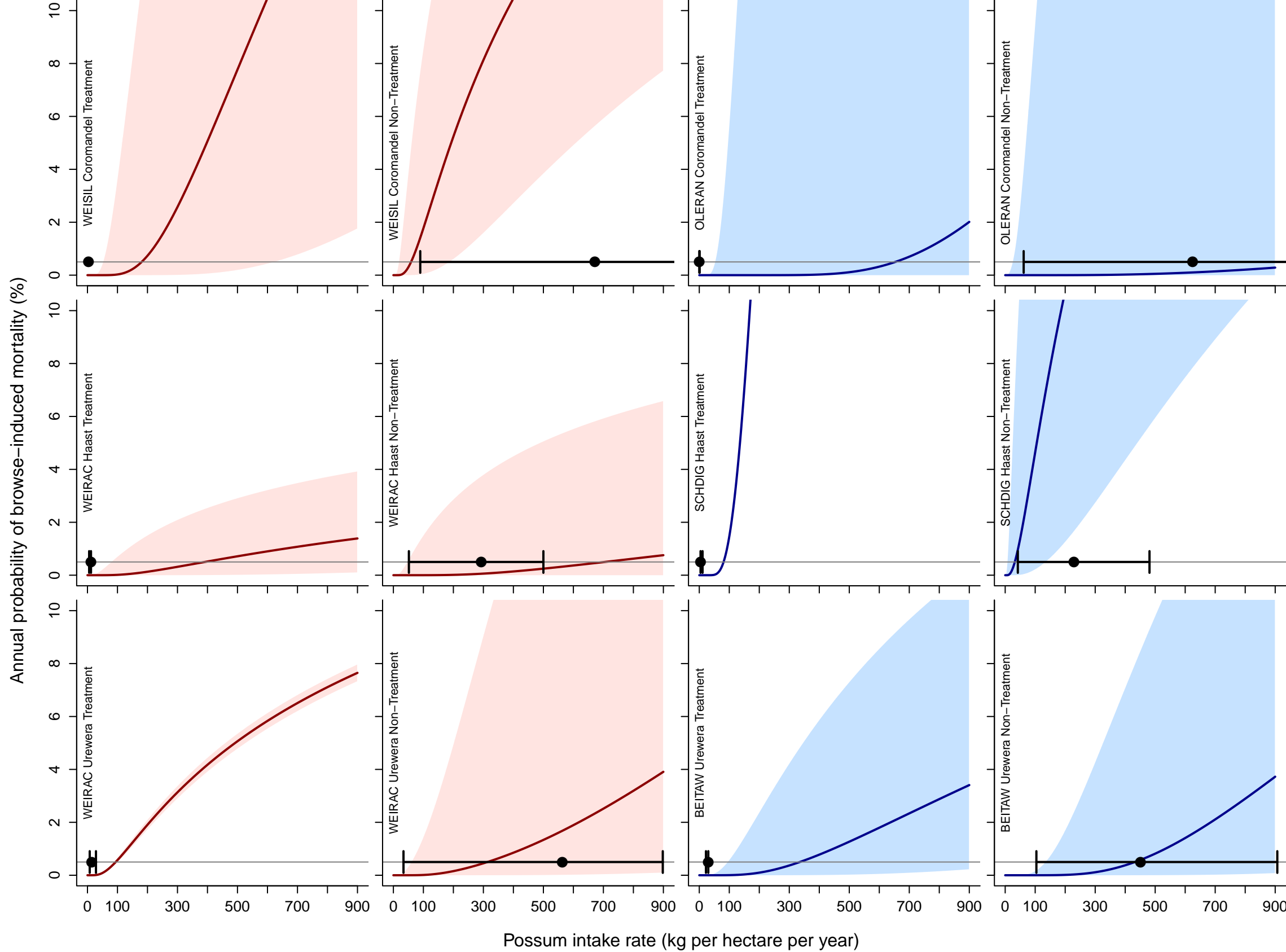

Supplement: Supplementary file 2 — Figure S2. Mean and 95% confidence intervals for predicted site‐wide annual probability of mortality of species + site combinations as a function of possum intake rate. [file ECE3-6-1954-s002.pdf]
