# Supplementary material for: Species‐ and site‐specific impacts of an invasive herbivore on tree survival in mixed forests
Source: Ecol Evol. 2016 Feb 24;6(7):1954–66. doi: 10.1002/ece3.2002 (PMC4767877; doi:10.1002/ece3.2002)
Supplement: Supplementary file 3 — Data S1. Additional investigation of how uncertainty in tree species and site level parameters in the browse model will affect results. Includes Tables S1 and S2. [file ECE3-6-1954-s003.doc]

Supporting Information
Species- and site-specific impacts of an invasive herbivore on tree survival in mixed forests.
Holland EP, Gormley AM, Pech RP
Ecology and Evolution

**Uncertainty in tree species parameters**

Parameters *a* and *e* were fitted to data from four tree species. Leaf life span *a* was estimated by fitting *A*, the asymptote of growth rate with increasing foliar mass/area remaining. The standard error of *A* was always small (in the region 0.001 - 0.004). We therefore did not consider further variation in *a* as being significant. The standard error of *e* was larger; this had a knock-on effect on the intake threshold (I'/B), intakes above which are considered to lead to death by possum browse. In fact, the upper limit of the 95% confidence interval (calculated as the 97.5% quantile of the normal distribution with mean and standard deviation given in Table S1) only increases the intake threshold by 2 - 7% of attainable biomass (B) (Table S1). The lower limit for *e* is calculated as the lowest value for which growth will be at least 0.95 *a* when the tree has not been browsed (i.e. the tree is likely to be able to maintain its attainable foliar biomass in an unbrowsed state). This results in an intake threshold of 0.07 - 0.09, or 7-9% of attainable biomass removed by possums per year, which is 10 - 15% less than the mean value. This suggests that mortality estimates predicted by the model using the mean value of *e* are relatively conservative. If the growth rate of trees is less affected by browse than estimated by our model, the intake threshold does not increase very significantly. However, if the growth rate of trees is more affected by browse than is considered by this model, there is a much higher possibility of mortality as a result of browse alone, as much lower intake rates (kg per kg per year) could result in a tree crossing the intake threshold and being totally defoliated.

**Table S1.** Quantification of uncertainty around tree species parameters.

| Species code | Number of data points | Standard error of A | Estimate of e | Standard error of e | Standard deviation of e | I'/B |
| --- | --- | --- | --- | --- | --- | --- |
| WEIRAC | 3995 | 0.0011 | 16.60 | 0.46 | 29.07 | 0.17 (0.07-0.21) |
| OLERAN | 2303 | 0.0016 | 14.54 | 0.55 | 26.39 | 0.22 (0.09-0.27) |
| SCHDIG | 1195 | 0.0039 | 60.79 | 3.58 | 123.76 | 0.23 (0.08-0.25) |
| BEITAW | 3047 | 0.0026 | 8.79 | 1.00 | 55.20 | 0.15 (0.07-0.22) |

**Uncertainty in site level parameters**

We calculated 95% confidence intervals for browse distribution parameters: foliage intake rate *I_s_* (kg dry weight ha^–1^ year^–1^), and browse preferences based on tree size (*y_s_*) and individual trees (*h_s_*) (Table S2). Mean values are given in Table 2 of the main text.

Uncertainty in the site-wide intake rate *I_s_* was relatively small at the treatment sites, with the largest confidence interval being for WEIRAC at the Urewera treatment site with a range of 7.48 - 28.3 kg ha year (Table S2). This is equivalent to 0.13 - 0.48 of one possum's diet (based on a yearly intake of 58.6 kg dry weight). The Urewera treatment site had the highest trap catch index (TCI) of the three treatment sites, with TCI = 4.9, which is approximately 0.26 possums per hectare (Ramsey et al. 2005). Since kamahi is unlikely to constitute the entire diet of any one individual possum, it seems likely that the true intake rate is closer to the lower end of the confidence interval, in which case using the mean values to estimate tree mortality will give very conservative management guidance.

Uncertainty in parameters for browse preference based on tree size (*y_s_*) and individual trees (*h_s_*) was relatively large at treatment sites (Table S2). This is a result of the data showing very few browsed trees (see the low and relatively certain intake rate reflected by values for *I_s_*), making it difficult to be certain about what strategy is being used to choose where to browse. However, since the intake rate is low, uncertainty in *y_s_* and *h_s_* is unlikely to affect model outputs significantly.

At the treatment sites, mean parameters for browse preference based on individual trees (*h_s_*) were usually closer to the lower end of the 95% confidence interval. This indicates a greater preference for individual trees (i.e. more non-uniform browse) is more likely. In contrast, the mean parameter for browse preference based on tree size (*y_s_*) was usually near the middle of the 95% confidence interval.

The estimated intake rate at non-treatment sites varied by an order of magnitude. This reflects the uncertainty in the data used to fit the parameters; estimating an intake rate in kg per kg attainable biomass involves estimating attainable biomass (as an allometric relationship from diameter at breast height), leaf mass and bite size, and relating these to observed, categorical indices of browse damage.

**Table S2.** Lower and upper limits of 95% confidence intervals for parameter values of foliage intake rate *I_s_* (kg dry weight ha^–1^ year^–1^), and browse preferences based on tree size (*y_s_*) and individual trees (*h_s_*)

|  | Site | Species code | *I_s_* | | *y_s_* | | *h_s_* | |
| --- | --- | --- | --- | --- | --- | --- | --- | --- |
|  |  |  | *2*·*5th* | *96*·*5th* | *2*·*5th* | *97*·*5th* | *2*·*5th* | *97*·*5th* |
| Coromandel | Treatment | WEISIL | 3·87 | 3·87 | 0·01 | 0·026 | 0·44 | 0·442 |
|  |  | OLERAN | 0·01 | 1·21 | 0·34 | 5 | 0·056 | 5 |
|  | Non-treatment | WEISIL | 89·6 | 1175 | 0·01 | 0·132 | 0·121 | 0·258 |
|  |  | OLERAN | 61·6 | 1252 | 2·75 | 4·07 | 0·402 | 1·019 |
| Haast | Treatment | WEIRAC | 5·60 | 11·7 | 1·10 | 1·76 | 0·01 | 0·589 |
|  |  | SCHDIG | 4·19 | 10·9 | 0·99 | 1·02 | 0·386 | 5 |
|  | Non-treatment | WEIRAC | 51·7 | 500 | 1·72 | 2·87 | 0·01 | 0·043 |
|  |  | SCHDIG | 42·1 | 481 | 2·02 | 2·79 | 0·185 | 0·345 |
| Urewera | Treatment | WEIRAC | 7·48 | 28·3 | 0·79 | 1·02 | 0·01 | 4·841 |
|  |  | BEITAW | 22·2 | 30·1 | 1·07 | 1·75 | 0·01 | 0·108 |
|  | Non-treatment | WEIRAC | 33·7 | 898 | 1·21 | 2·25 | 0·357 | 5 |
|  |  | BEITAW | 104 | 907 | 1·42 | 1·95 | 0·257 | 0·623 |

**Figure S1.** Annual probability of tree mortality attributable to possum browse predicted by the browse model (mean and 95% confidence intervals indicating mortality of small, average and large trees; solid line and shaded area respectively) as a function of Foliage Cover Index, compared with an hierarchical model of total observed mortality fitted to field data (circles; Gormley et al. 2012), as per Fig. 1 in the main text. Two additional mean and confidence interval combinations are also shown indicating a minimum damage scenario (low intake with uniform distribution across all trees, in dotted lines, mean shown by the thicker line) and a maximum damage scenario (high intake with preferential browse on a few, larger individuals, in dashed lines, mean shown by the thicker line). These scenarios use parameter values at the limits of 95% confidence intervals for parameters indicating browse preference (*h_s_*, *y_s_*) and intake rate (*I_s_*), rather than the mean. The minimum damage scenario usually indicates that no mortality at the site may be due to possum browse (confidence interval for probability of browse-induced mortality includes zero for all foliage cover indices), while the maximum damage scenario suggests that browse-induced mortality has a marginally higher mean and confidence interval compared to the mean damage scenario. The impact of possum browse on annual probability of mortality does not change greatly for trees of average size (thicker lines) at any of the sites.

**Figure S2.** Mean and 95% confidence intervals for predicted site-wide annual probability of mortality of species+site combinations as a function of possum intake rate. The grey horizontal line indicates site-wide mortality equal to 0.5% per species per year. The black circle and attached arrows indicate the mean and 95% confidence interval for possum intake rate at the site at Time 1. These are in contrast to the single estimate of possum abundance (trap catch index; TCI) obtained at Time 1 and displayed on Fig. 2 in the main text. Intakes high enough to cause tree mortality over the 0.5% management threshold are clearly evident at the non-treatment sites despite the wide confidence intervals, but not at those that had experienced treatment (possum control).
